# Using preoperative ultrasound vascularity characteristics to estimate medullary thyroid cancer

**DOI:** 10.1186/s40644-023-00583-6

**Published:** 2023-06-20

**Authors:** Luying Gao, Liyuan Ma, Xiaoyi Li, Chunhao Liu, Naishi Li, Xiaolan Lian, Weibo Xia, Ruifeng Liu, Xinlong Shi, Jiang Ji, Aonan Pan, Yu Xia, Yuxin Jiang

**Affiliations:** 1 Department of Ultrasound, State Key Laboratory of Complex Severe and Rare Diseases, Peking Union Medical College Hospital, Chinese Academy of Medical Sciences and Peking Union Medical College, 100730 Beijing, China; 2 Department of General Surgery, State Key Laboratory of Complex Severe and Rare Diseases, Peking Union Medical College Hospital, Chinese Academy of Medical Sciences and Peking Union Medical College, Beijing, China; 3 Department of Endocrinology, State Key Laboratory of Complex Severe and Rare Diseases, Peking Union Medical College Hospital, Chinese Academy of Medical Sciences and Peking Union Medical College, Beijing, China

**Keywords:** Medullary thyroid carcinoma, Ultrasound, Thyroid carcinoma

## Abstract

**Background:**

The early diagnosis of medullary thyroid carcinoma (MTC) is still a challenge in clinical practice. Based on ultrasound features, many MTC cases without suspicious characteristics are not categorized as high risk for malignancy. This study was designed to comprehensively investigate the ultrasonic features of MTC on ultrasound and help identify thyroid nodules with a high risk of MTC.

**Methods:**

Between 2017 and 2023, we retrospectively reviewed 116 consecutive thyroid nodules with a histologic diagnosis of MTC who had undergone preoperative ultrasound examination. According to the ultrasonic criteria for risk classification, nodules were classified as “ultrasound-high suspicious” (h-MTC) and “ultrasound-low suspicious” (l-MTC). Using the same database, a tumour size- and risk evaluation-matched control group comprising 62 lesions was randomly selected to compare the vascularity features of l-MTC disease.

**Results:**

We identified 85 h-MTC nodules (73.3%) and 31 l-MTC nodules (26.7%). For patients with l-MTC disease, 22/31 (71.0%) of the lesions were followed up for a period before fine needle aspiration (FNA) or surgery. We observed more penetrating branching vascularity in the l-MTC group than in the benign nodule group (23/31, 74.2% vs. 5/59, 4.8%, P < 0.001). We also showed that more CHAMMAS IV patterns (central blood flow greater than perinodular flow) (87.1% vs. 32.3%, P < 0.001)) and CHEN IV patterns (penetrating vascularity) (100% vs. 25.8%, P < 0.001) were found in l-MTC than benign nodules.

**Conclusions:**

Vascularity features can help differentiate l-MTC from benign nodules; moreover, we report a novel sonographic vascularity pattern of l-MTC disease, penetrating branching vascularity. The utilization of vascularity features will help to identify MTC among nodules with low-intermediate suspicion by ultrasound risk classification to ensure appropriate clinical management.

**Supplementary Information:**

The online version contains supplementary material available at 10.1186/s40644-023-00583-6.

## Background

Medullary thyroid carcinoma (MTC) originates from C cells and accounts for 5–8% of thyroid malignancies [1]. MTC is more prone to lymph node (LN) and distant metastases, and up to 13% of thyroid cancer-related deaths are caused by MTC [2, 3]. It has been notoriously difficult to cure if not diagnosed early on. The primary imaging tool in the diagnosis of MTC is ultrasound, but ultrasound also fails to achieve high reliability with a sensitivity of 75% [1, 4, 5]. There are also several defects in fine needle aspiration (FNA) of MTC, and the detection rate was only 56% in a meta-analysis [2]. Calcitonin is a valuable marker for MTC and is more specific and sensitive than FNA for diagnosing MTC [6]. Several studies have demonstrated the usefulness of calcitonin in the diagnostic work-up of thyroid nodules [5, 7, 8]. In a series where > 10,000 patients with thyroid nodules underwent calcitonin testing, 0.4% had an elevated serum calcitonin level [8]. However, the direct screening measurement of serum calcitonin in all nodules with benign ultrasonic presentation is still a matter of debate due to the low prevalence of the disease [1]. Because of these limitations, many MTCs are still found incidentally after thyroidectomy, leading to the risk of incomplete treatment and thus of a poorer prognosis [9, 10].

Ultrasound (US) is the preferred method for not only discriminating between benign and malignant thyroid lesions but also as guidance for FNA. In many established guidelines, nodules have been stratified on the basis of suspicious ultrasound characteristics [11–13]. However, many MTC nodules were not stratified by any system as having characteristics indicating a high risk of malignant tumours, and because of large size thresholds, fewer biopsies have been recommended [14, 15]. Matrone et al. showed that FNA was only recommended for 49–64% of MTC nodules. Many MTCs without suspicious ultrasound features are not categorized as high risk of malignancy [15]. Vascularity, as an important characteristic of MTC, was not included in many societal US risk stratification systems [11]. Previous studies have also shown that hypervascularity is more frequent in MTC by comparing its US features with those of papillary thyroid cancer [16, 17]. In this regard, our purpose with this study was to more fully investigate the vascularity characteristics of MTC on ultrasound and help screen nodules to identify MTC.

### Patients and methods

This study was approved by the ethics committee of our centre (JS-2881). All procedures performed in the studies were in accordance with the 1964 Helsinki declaration and its later amendments or comparable ethical standards. Written informed consent for participation was not required for this study in accordance with national legislation and institutional requirements.

### Patient identification

Between January 2017 and April 2023, a total of 8709 patients who underwent thyroidectomy at our centre were retrospectively identified. During the initial evaluation, only lesions that met the following criteria were included: (a) thyroidectomy had been performed during the first surgery; (b) MTC confirmed by histological examination according to the WHO classification criteria [18]; (c) age > 18 years old; and (d) preoperative sonographic findings were available. A total of 116 MTC nodules in 102 patients met the inclusion criteria.

According to the ultrasonic criteria for risk classification of the American Thyroid Association (ATA), nodules with suspicious ultrasound features were categorized as “ultrasound-high suspicious” (h-MTC), and those without suspicious ultrasound features were categorized as “ultrasound-low suspicious” (l-MTC). The h-MTC category included the ATA high suspicion pattern, and the l-MTC category included the ATA patterns of intermediate suspicion, low suspicion, very-low suspicion, and benign. Eighty-five lesions were classified as h-MTC, and 31 lesions were classified as l-MTC. Those with l-MTC could be misdiagnosed as being benign lesions. The ultrasound characteristics of l-MTC disease were compared with those of benign lesions. Using the same database, patients who met the following criteria were included: (a) thyroidectomy performed during the first surgery due to pathologically confirmed benign lesions; (b) classified as low-intermediate suspicion by ultrasound ATA pattern; and (c) age > 18 years old. A tumour size-matched control group comprising 62 lesions was randomly selected.

### Data collection

Ultrasound examinations were performed with an IU 22 or EPIQ 7 (Philips Medical Systems, Bothell, WA) system equipped with a 5–12 MHz linear transducer. The tumour location, composition, echogenicity, shape, border, microcalcifications, ATA risk stratification, and vascularity of thyroid nodules were evaluated by retrieving and reviewing the thyroid ultrasound images in the PACS systems. Thyroid nodules were stratified into five risk stratifications following the 2015 ATA guidelines (high suspicion, intermediate suspicion, low suspicion, very-low suspicion, and benign) [11].

**Fig. 1:**
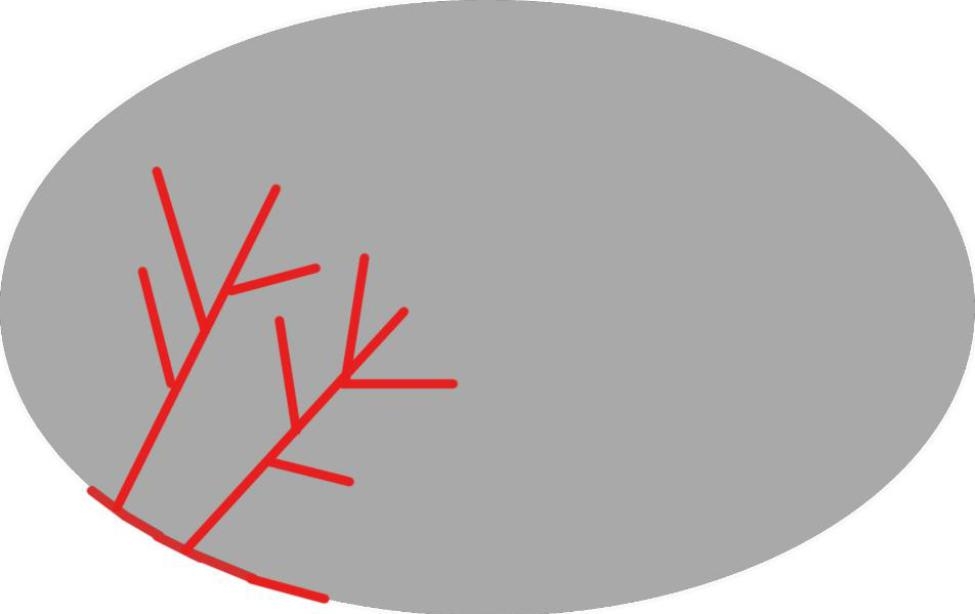
Schematic of penetrating branching vascularity based on the distribution patterns of nodule vascularity on ultrasound. Penetrating branching vascularity: branching signals extending from the outside flow to the inside of the lesion

Colour Doppler analysis of the nodules was classified into the following CHAMMAS patterns: I, no signal blood flow; II, exclusively perinodular vascularity; III, perinodular blood flow ≥ central blood flow; IV, marked central blood flow and less marked perinodular blood flow; and V, exclusively central vascularity [19]. According to the method recommended by CHEN et al., nodular vascularity on colour Doppler can be divided into four types: type I, no vascularity; type II, mainly perinodular vascularity with continuous (IIa) or discontinuous peripheral vascularity blood vessels (IIb); type III, mainly intranodular vascularity, linear (IIIa), branching (IIIb) or diffusing (IIIc), with or without perinodular vessels; and type IV, penetrating vascularity with (IVa) or without (IVb) perinodular vascularity of a nodule [20]. Penetrating branching vascularity was defined as branching signals extending from the flow from the outside to the inside of the lesion [Fig. 1]. Two radiologists with 5 and 7 years of experience in thyroid imaging reviewed the images in the mixed dataset with other diagnoses (benign nodules). Reviewers were blinded to the patients’ clinical data, pathological results, and each other’s evaluations. The inconformity was resolved by a radiologist with 21 years of experience in thyroid imaging.

### Statistical analysis

For parametric data, an unpaired t test was used to evaluate the difference between the two groups. For nonparametric data, the Mann‒Whitney U test was used to analyse the differences between groups. The chi-square test with Yates’ correction and Fisher’s exact test were used to compare the categorical variables. All statistical analyses were performed using SPSS software version 22.0 (IBM, Armonk, NY, USA). A difference of P < 0.05 was considered statistically significant.

## Results

### The clinical and biochemical characteristics of patients with MTC

The present study consisted of 116 thyroid nodules in 102 patients with pathologically proven MTC lesions, of which 88 patients had a single MTC nodule and the other 14 patients presented with 2 MTC nodules. The patients were predominantly female (60/102, 58.8%). The mean age was 47.6 ± 15.5 years (range, 18–84 years). Three cases (2.6%) were MEN 2. According to the ultrasonic criteria for risk classification, 85 nodules (73.3%) were classified as h-MTC (high suspicion by ATA classification) and 31 nodules (26.7%) as l-MTC [(intermediate suspicion by ATA classification (n = 22, 71.0%); low suspicion by ATA classification (n = 9, 29.0%)]. There was no statistically significant difference in age (p = 0.078), sex (p = 0.38), preoperative Ct level (p = 0.88), or preoperative CEA level (p = 0.75) between the two groups. Moreover, patients in the l-MTC group had a larger tumour size than those in the h-MTC group (p < 0.001) [Table 1]. All l-MTC nodules were initially found by US. Since they were classified as intermediate/low suspicion by the ultrasound risk evaluation, 22/31 (71.0%) of the lesions were followed up for a period before FNA or surgery. Of the 31 cases with l-MTC, the indication for FNA or surgery included suspicious lymph node metastasis on preoperative ultrasound (14, 45.2%); enlarged tumour size (8, 25.8%); and measurement of serum CEA/CT level due to suspicious MTC features (increased intranodular hypervascularity) on ultrasound (9, 29.0%). Of the 85 cases of h-MTC, all patients underwent FNA or surgery due to high suspicion of malignant thyroid nodules on preoperative ultrasound.

**Table 1:** Comparison of clinical characteristics

Variable	l-MTC	h-MTC	P
No.	31	85	
Age, y (median)	48.00	47.45	0.078
Sex (female/male)	17/14	51/34	0.38
Tumour size (cm)	2.15	1.37	< 0.001
Preoperative Ct level (pg/mL)	416.80	443.58	0.88
Preoperative CEA level (pg/mL)	43.98	38.23	0.75

Abbreviations: No., number; y, years old; Ct, calcitonin; l-MTC, ultrasound-low suspicious medullary thyroid carcinoma; h-MTC, ultrasound-high suspicious medullary thyroid carcinoma

### Blood supply characteristics on US and differences between the L-MTC group and the benign group

**Fig. 2:**
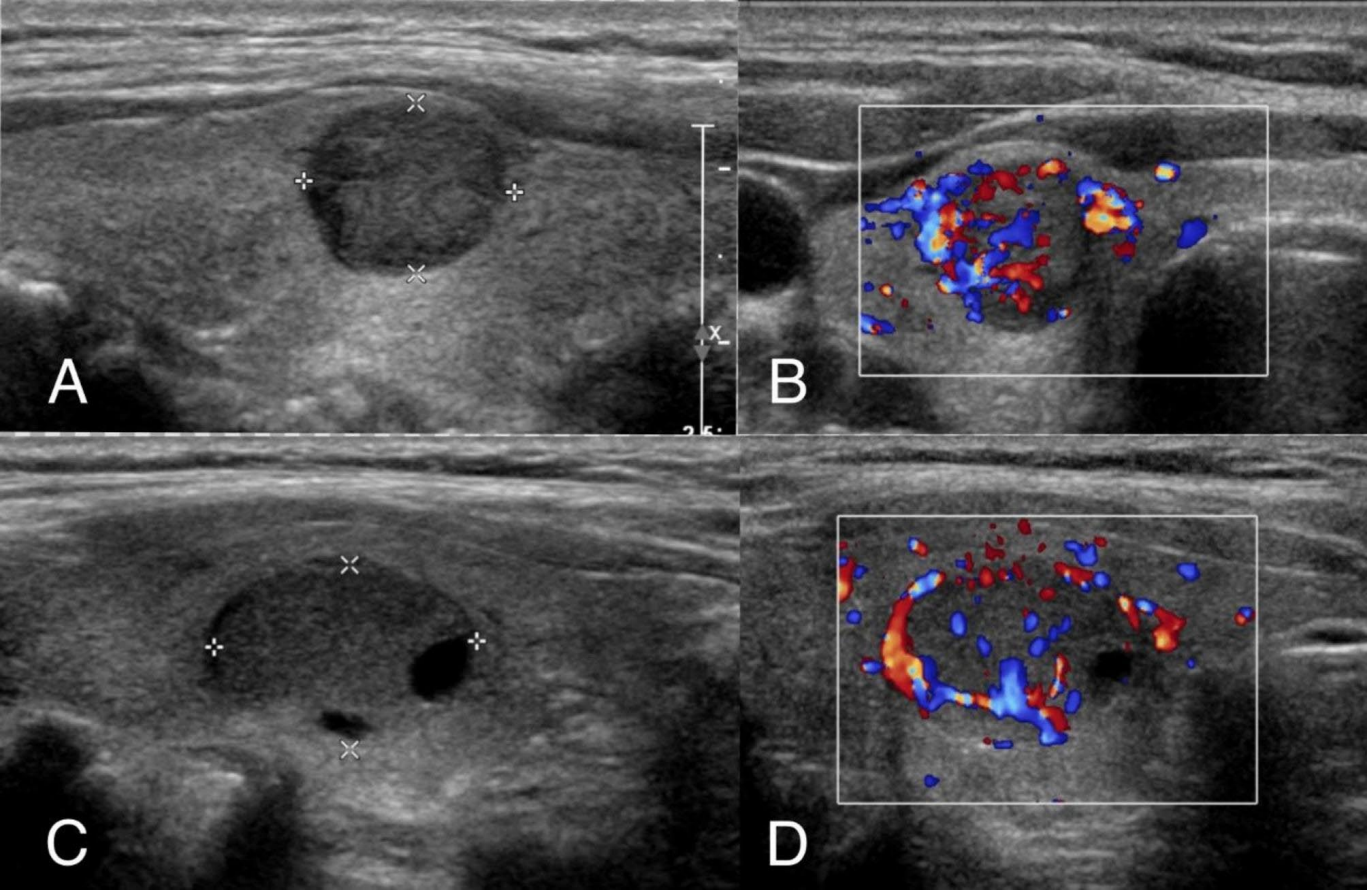
Two examples illustrating the vascularity features of penetrating branching vascularity. (**A**) A case of a 57-year-old man whose ultrasound showed a 1.2-cm solid thyroid nodule in the right lobe. (**B**) CDFI of the lesion. (**C**) A case of a 60-year-old woman whose ultrasound showed a 1.6-cm solid thyroid nodule in the right lobe. (D) CDFI of the lesion. Histological pathology confirmed that these two nodules were MTC.

Regarding the echogenicity of the nodules, 11 (35.5%) were solid, 20 (64.5%) had a mixed structure, 26 (83.9%) were hypoechoic, and 5 (16.1%) were isoechoic/hyperechoic. In the present study, we compared the blood supply characteristics on US of l-MTC disease with 62 benign lesions from 62 patients. According to the blood flow pattern of CHAMMAS, 4 (12.9%) l-MTC disease had perinodular blood flow greater than central flow (pattern III), and 27 (87.1%) l-MTC disease had central blood flow greater than perinodular flow (pattern IV). Statistical analysis revealed a significant relationship between pattern IV and l-MTC (P < 0.05). With respect to the CHEN pattern of vascularity distribution, 31 (100.0%) were type IV for the l-MTC, 14 (22.6%) were type I, 19 (30.6%) were type II, 13 (21.0%) were type III and 16 (25.8%) were type IV for the benign nodules. More penetrating branching vascularity was found in the l-MTC group than in the benign nodule group (23/31, 74.2% vs. 5/59, 4.8%, P < 0.001). Thus, type IV blood flow patterns were more frequent in the L-MTC group (P < 0.05) [Table 2; Fig. 2].

**Table 2:** Sonographic characteristics of the l-MTC group compared with the benign group

Variable	l-MTC	Benign Group	P
Age, y (median)	48.00	54.61	0.08
Sex (female/male)	17/14	14/28	0.88
Tumour size (cm)	2.15	2.07	0.79
Echogenicity			0.30
Hyperechogenicity/Isoechogenicity	5 (16.1%)	16 (25.8%)	
Hypoechogenicity	26 (83.9%)	46 (74.2%)	
Cystic change (%)	20 (64.5%)	29 (46.8%)	0.10
ATA classification			0.06
Intermediate Suspicion	22 (71.0%)	31 (50.0%)	
Low Suspicion	9 (29.0%)	21 (33.9%)	
Very low Suspicion	0	10 (16.1%)	
Vascularity			
CHAMMAS pattern ^[15]^			< 0.001
Pattern II	0	14 (22.6%)	
Pattern III	4 (12.9%)	28 (45.2%)	
Pattern IV	27 (87.1%)	20 (32.3%)	
CHEN pattern ^[16]^			< 0.001
Pattern I	0	14 (22.6%)	
Pattern II b	0	19 (30.6%)	
Pattern III c	0	13 (21.0%)	
Pattern IV a	31 (100%)	16 (25.8%)	
Penetrating branching vascularity			< 0.001
YES	23 (74.2%)	5 (4.8%)	
NO	8 (25.8%)	59 (95.2%)	

Abbreviations: y, years old; l-MTC, ultrasound-low suspicious medullary thyroid carcinoma; ATA, American Thyroid Association

## Discussion

The present results showed that approximately 1/3 of MTCs were not categorized as high risk of malignancy based on societal US guidelines, which led to most of these MTC lesions being followed up for a period before treatment in clinical practice. As vascularity is an important characteristic of l-MTC disease, we discerned a sonographic blood supply feature, penetrating branching vascularity, that identified more than 70% of l-MTC in our series. Only 5% of our cases with benign nodules displayed penetrating branching vascularity features, supporting that this feature is a valuable sign of l-MTC. We also showed that more CHAMMAS IV patterns (central blood flow greater than perinodular blood flow) (89% vs. 39%) and CHEN IV patterns (penetrating vascularity) (100% vs. 44%) were found in l-MTC disease than benign nodules.

Ultrasound is first-line imaging for patients with suspected MTC. Researchers have evaluated the sonographic features of MTCs in several studies, and US is associated with a sensitivity of 75% and a specificity of 93% [21]. In all these studies, it was reported that MTCs may not have classical ultrasonic malignant features of thyroid nodules, which include irregular shape, height/width ratio < 1, and microcalcification. [22–24] In particular, patterns of benign features are often recorded in MTC, mainly including solid, oval to round shapes and smooth margins. Previous studies have shown that approximately one-third of MTC patients manifest as benign on ultrasound, which is very similar to the situation indicated by our data [6]. Although one study showed that the ATA guidelines performed well in the prediction of MTC [2], more studies and our data revealed that approximately 27-47% of MTC cases were not classified by any of the systems as suggestive of a high risk of malignancy [15]. According to the ultrasonic criteria for risk classification, l-MTCs were misdiagnosed as such and were not treated early on. In our cases, most patients with those lesions (71%) were followed up for a period rather than given immediate treatment. Among our patients with l-MTC disease, indications for FNA or surgery included suspicion of lymph node metastasis on ultrasound, increased tumour volume, and serum CEA/CT levels. Calcitonin is more specific and sensitive than FNA for diagnosing MTC, while clinical screening of serum calcitonin is still not widely applied due to the low prevalence of the disease [1].

In addition to morphology, vascularity is also an important factor in the differential diagnosis of lesions. According to two previous studies, increased intranodular hypervascularity was more frequent in MTCs [16, 17]. Our team’s previous study showed that hypervascularity is more frequent in medullary thyroid carcinoma by comparing it US features with those of papillary thyroid cancer [16]. However, Trimboli et al. [25] reported that 25% of MTCs exhibited an intranodular vascular signal. This discrepancy might be explained by the small sample size in the study and the inclusion of more h-MTCs. To the best of our knowledge, reports of the vascular pattern of l-MTCs in the literature are limited. In this study, we discovered the usefulness of penetrating branching vascularity in distinguishing l-MTCs. Penetrating branching vascularity, branching signals that extend from the flow of outside to the inside of the lesion, are different from intranodular vessels. The occurrence, development, and invasion of tumours highly depend on angiogenesis, which refers to the formation of new blood vessels through “endothelial cell germination” from the original microvascular network. Folkman et al. proved that proangiogenic factors generated from masses stimulated new blood vessels converging on lesions from some distance [26]. In our study, more than 70% of l-MTC cases manifested penetrating branching vascularity, demonstrating the value of penetrating branching vessels in the diagnosis of l-MTC disease. Moreover, the value of penetrating vessels in the diagnosis of malignant nodules was previously reported with the finding that thyroid cancer manifested penetrating vascularity [20, 27]. Chen et al. showed that 40% (17/43) of malignant thyroid nodules manifested penetrating vascularity and that this feature could improve the diagnostic performance of TI-RADS for TR4 thyroid nodules [20]. Researchers at our centre also found previously that penetrating vessels around the lesion are useful for determining thyroid carcinoma [27].

The main limitation of this research was the relatively small number of patients in the retrospective study. Given the retrospective nature of the study, radiologists were not blinded, and static images were utilized during the review process. Bias may exist, and static images may not adequately reflect the status of the nodule. Prospective studies with larger sample sizes are necessary to validate our proposed vascularity feature. Second, this was a single-centre study. There might have been selection bias. Future research should expand on our observations to determine if the described pattern appears in other pathologies and its rate of occurrence.

## Conclusion

Based on societal US guidelines, some MTCs are mistakenly not categorized as high risk of malignancy, and vascularity can help diagnose l-MTC disease. We report a novel sonographic vascularity pattern of l-MTC, i.e., penetrating branching vascularity. The utilization of vascularity will help to identify MTC among nodules with low-intermediate suspicion by ultrasound risk classification to ensure appropriate clinical management.

## Electronic supplementary material

Below is the link to the electronic supplementary material.

Supplementary Material 1

Supplementary Material 2

## Data Availability

The datasets generated during and/or analysed during the current study are available from the corresponding author on reasonable request.

## Abbreviations

MTC: Medullary thyroid carcinoma
h-MTC: Ultrasound-high suspicious medullary thyroid carcinoma
l-MTC: Ultrasound-low suspicious medullary thyroid carcinoma
No.: Number
CI: Confidence interval
y: Years old
ATA: American Thyroid Association
Ct: Calcitonin
US: Ultrasound
FNA: Fine needle aspiration
