# Supplementary material for: Using preoperative ultrasound vascularity characteristics to estimate medullary thyroid cancer
Source: Cancer Imaging. 2023 Jun 20;23:64. doi: 10.1186/s40644-023-00583-6 (PMC10280910; doi:10.1186/s40644-023-00583-6)
Supplement: Supplementary file 1 — Supplementary Material 1 [file 40644_2023_583_MOESM1_ESM.docx]

**Response to the reviewers**

**Manuscript Number: CAIG-D-22-00417**

**Manuscript Title: Using Preoperative Ultrasound Vascularity Characteristics to Estimate Medullary Thyroid Cancer**

Dear Reviewers and editors,

Thank you very much for all of your work on our manuscript, which we really appreciate. The manuscript has now been revised to address the comments and suggestions point by point. In the following statements, we will introduce what we have revised and modified during the revision following each comment.

**Reviewer #1: In the present paper, Gao and colleagues investigated the vascularization of medullary thyroid cancer on ultrasound to verify if it can help the identification of this neoplasm.
The topic is very interesting, because the role of thyroid ultrasound and the ability of several classification to detect medullary thyroid cancer are still debated; however, none of the classifications currently used take vascularity into consideration. Therefore, this could be a useful and effective tool, indeed Gao's results allow us to identify a vascular pattern that distinguishes benign nodules from low-risk medullary thyroid cancer, that have more probability of being misdiagnosed.
Major comments
Line 57 - The authors reported that routine testing of calcitonin is still controversial. Although this approach is debated, several evidence showed that the routine evaluation of calcitonin in the diagnostic work-up of thyroid nodules led to the discover of less aggressive MTC with higher probability of cure. Therefore, the authors should complete the sentence specifying that "however, several papers clearly demonstrated the usefulness of the routine use of calcitonin in the diagnostic work-up of thyroid nodules". Moreover, they should specify in the introduction and discussion section that, at now, calcitonin is the best tool in the diagnosis of MTC (PMID 35422765, 19041824, 14715844).**

Response: Thank you for your positive comments. We followed the suggestions, “Calcitonin is a valuable marker for MTC, and is more specific and sensitive than FNA for diagnosing MTC” (PMID: 34659736) and “Several papers demonstrated the usefulness of calcitonin in the diagnostic work-up of thyroid nodules (PMID 35422765, 19041824, 14715844)” and “In the series where > 10,000 patients with thyroid nodule underwent calcitonin test, 0.4% had an elevated serum calcitonin level (14715844)” has been added to the introduction and reference section and highlighted in red color (Lines 50-53).

**Lines 130-131 - The authors reported that I-MTC had higher tumor size compared with h-MTC, however no difference in the calcitonin (and CEA) values have been observed. The authors should widely discuss why I-MTC were larger than h-MTC in their series, but mostly why no difference in calcitonin levels were observed, since it is known that larger MTC dimension are associated to higher calcitonin values. In table 1, the median tumor dimension was very different. Please clarify.**

**Response:** Thank you for your comments. Cases in I-MTC group did have a larger tumor size than that in h-MTC (2.15 cm v s 1.37 cm, p<0.001). In clinical practice, according to FNA indication of the ultrasonographic risk stratification systems, all cases with h-MTC undergo immediate FNA or surgery due to high suspicion of malignancy on ultrasound. According to the ultrasonic criteria for risk classification, those with l-MTC may be misdiagnosed as suspicion of benign lesions and were not diagnosed early on. Since l-MTC was classified as intermediate/low suspicion of the ultrasound risk evaluation, most (22/31,71.0%) of the lesions were followed up for a period rather than undergoing immediate FNA or other interventions. Among the 31 patients with l-MTC, indications for FNA or surgery included suspicion of lymph node metastasis on preoperative ultrasound (14, 45.2%); increased tumor volume (8, 25.8%); and measurement of serum CEA/CT levels (9, 29.0%).

As to our data, cases in l-MTC group had a larger MTC dimension than cases in h-MTC group, however were not associated to higher baseline serum calcitonin values. One major reason may be the relatively small number of cases in the single-center study, and baseline serum Ct was only available in 20 cases in I-MTC group (63%), 51 cases in h-MTC group. Moreover, previous study showed that cases with US-low-suspicious has a more indolent character than those with high-suspicious, which may partly explain the level of serum Ct [1].

[1] Zhao J, Zheng X, Gao M, Zhang S, Yun X, Chi J, Xu G. Ultrasound features of medullary thyroid cancer as predictors of biological behavior. Cancer Imaging. 2021;21:33.

**Lines 126-128 - The authors classified the cases as h-MTC or as l-MTC using the ATA classification, although it is not shown on page 2 (line 86). However, on page 4 (lines138-139) the authors use another classification (ACR-TIRADS) for I-MTC. Why do they use two different ultrasound classifications at two different moments of the analysis? I suggest using only one in both analysis (also because it is already reported on page 3 line 128)**

**Response:** Thank you for your comments. We added following sentence to the methods section and highlighted in red color (Lines 78-82). “According to the ultrasonic criteria for risk classification of the American Thyroid Association (ATA), nodules with suspicious ultrasound features were categorized as “ultrasound-high suspicious” (h-MTC), and those without suspicious ultrasound features were categorized as “ultrasound-low suspicious” (l-MTC). The h-MTC category included the ATA high suspicion pattern, and the l-MTC category included the ATA patterns of intermediate suspicion, low suspicion, very-low suspicion, and benign.”

We followed the suggestions and deleted the content of ACR-TIRADS ultrasound classifications in the methods and results section (Line 103; Lines 136; Table 2).

**Page 4 lines 134-135 - measurement of serum calcitonin was performed due to suspicious MTC features; authors should specify what these characteristics are.**

**Response:** Suspicious MTC features are “increased intranodular hypervascularity”, which we added in the results section and highlighted in red color (Lines 123).

[1] Trimboli P, Nasrollah N, Amendola S, Rossi F, Ramacciato G, Romanelli F, Aurello P, Crescenzi A, Laurenti O, Condorelli E, Ventura C, Valabrega S. Should we use ultrasound features associated with papillary thyroid cancer in diagnosing medullary thyroid cancer? Endocr J. 2012;59:503-8.

**Page 4 lines 149-155 - the paragraph "tumor size, lymph node, and biochemical cure by basal calcitonin" seems written in a very hurried way, without providing concrete data and without referring to table 3. In Table 3, besides the descriptive data, it could be useful to report the statistical significance of the differences. The authors do not specify what they mean by large-volume of lymph node (what is the dimensional cut off? or do they consider lymph nodes that are not micro metastases?), and they do not reported after how long to follow up they define biochemical remission.
Final consideration
Finally, in my opinion, since several issues about this paper derived from the reports of biochemical data (calcitonin and CEA), since in not all patients this data is available and since the paper is focused on US features and vascularization and the biochemical information in this way are secondary, my suggestion is to delete these information and focus the attention on US and vascularization features, the main aims of the paper.**

Response: To the above two questions, we followed the suggestions and deleted the content of biochemical data in the abstract, introduction, methods, results and discussion section (Line 38, Line 78, Line 113; Line 140; Lines 178; Table 3) and focus on ultrasound and vascularization features.

**Minor comments
The paper should be revised by an English native writer.**
Response: We carefully reviewed the language, especially on the method and discussion parts, and sent our manuscript to English native experts from AJE (https://secure.aje.com/researcher/) for English-language improvement (An Editing Certificate has been attached). All inadequate scientific language has been corrected by experts and highlighted in red (Line 26, Line 33, Line 39; Line 73-74; Lines 151-153; Lines 140-142; Lines 146; Lines 148-149; Lines 160-163; Lines 164; Lines 171-172; Lines 178-179; Lines 183-184,etc).

**Reviewer #2: The present manuscript reported that vascularity features on ultrasound could help differentiate medullary thyroid carcinoma (MTC) from benign nodules, especially in those with low-intermediate suspicion of ultrasound risk classification. Preoperative serum calcitonin levels did not show a successive involvement of lymph node compartments. I am convinced that proposed research is a beneficial contribution to current scientific discussion on thyroid cancer diagnosis, since the MTC is a rare form of thyroid cancer. However, I am afraid that the proposed paper is relatively brief and authors should provide more details on some parts of the paper, especially methods, to make the research better understandable and reproducible.**
Response: Thank you for your positive comments. To be more successive and understandable, we deleted the content of biochemical data and focus on ultrasound and vascularization features. We added following sentence to the methods section “According to the ultrasonic criteria for risk classification of the American Thyroid Association (ATA), nodules with suspicious ultrasound features were categorized as “ultrasound-high suspicious” (h-MTC), and those without suspicious ultrasound features were categorized as “ultrasound-low suspicious” (l-MTC). The h-MTC category included the ATA high suspicion pattern, and the l-MTC category included the ATA patterns of intermediate suspicion, low suspicion, very-low suspicion, and benign.”, which are highlighted in red color (Lines 78-82).

**Other comments:
Abstract
- Please clarify the purpose of this study.
- The authors should mention role of "retrospective review".
- Line 31-32, should read "Based on ultrasound features, many MTC cases without suspicious characteristics are not categorized as high risk for malignancy".
- Line 33, "medullary thyroid carcinoma (MTC)" should be "MTC".
- Line 37, "I-MTC" should be "l-MTC".
- Line 40, should read "We identified 51 h-MTC (66.4%) and 18 l-MTC (33.6%)".
- Line 46, should read "there was no significant difference in successive involvement of lymph node compartments (P>0.05)".**
Response: Thank you for your comments and careful modifications. We followed all the suggestions and highlighted in red color (Lines 25-34; Lines 28; Lines 33). Abstract sections are as follows.

Abstract

*Background:* The early diagnosis of medullary thyroid carcinoma (MTC) is still a challenge in clinical practice. Based on ultrasound features, many MTC cases without suspicious characteristics are not categorized as high risk for malignancy. This study was designed to comprehensively investigate the ultrasonic features of MTC on ultrasound and help identify thyroid nodules with a high risk of MTC.

*Methods:* Between 2017 and 2023, we retrospectively reviewed 116 consecutive thyroid nodules with a histologic diagnosis of MTC who had undergone preoperative ultrasound examination. According to the ultrasonic criteria for risk classification, nodules were classified as “ultrasound-high suspicious” (h-MTC) and “ultrasound-low suspicious” (l-MTC). Using the same database, a tumour size- and risk evaluation-matched control group comprising 62 lesions was randomly selected to compare the vascularity features of l-MTC disease.

*Results:* We identified 85 h-MTC nodules (73.3%) and 31 l-MTC nodules (26.7%). For patients with l-MTC disease, 22/31 (71.0%) of the lesions were followed up for a period before fine needle aspiration (FNA) or surgery. We observed more penetrating branching vascularity in the l-MTC group than in the benign nodule group (23/31, 74.2% vs. 5/59, 4.8%, P<0.001). We also showed that more CHAMMAS IV patterns (central blood flow greater than perinodular flow) (87.1% vs. 32.3%, P<0.001)) and CHEN IV patterns (penetrating vascularity) (100% vs. 25.8%, P<0.001) were found in l-MTC than benign nodules.

*Conclusions:* Vascularity features can help differentiate l-MTC from benign nodules; moreover, we report a novel sonographic vascularity pattern of l-MTC disease, penetrating branching vascularity. The utilization of vascularity features will help to identify MTC among nodules with low-intermediate suspicion by ultrasound risk classification to ensure appropriate clinical management.

**Background
- To make the background more substantial, the author should add several updated references to substantiate the claim made.**
Response: Thank you for the comments. As your following suggestions, we expand the background part and add more references as follows, which were highlighted in red color (Lines 30-34; Lines 35; Lines 40).

Background

Medullary thyroid carcinoma (MTC) originates from C cells and accounts for 5% to 8% of thyroid malignancies [1]. MTC is more prone to lymph node (LN) and distant metastases, and up to 13% of thyroid cancer-related deaths are caused by MTC [2, 3]. It has been notoriously difficult to cure if not diagnosed early on. The primary imaging tool in the diagnosis of MTC is ultrasound, but ultrasound also fails to achieve high reliability with a sensitivity of 75% [1,4,5]. There are also several defects in fine needle aspiration (FNA) of MTC, and the detection rate was only 56% in a meta-analysis [2]. Calcitonin is a valuable marker for MTC and is more specific and sensitive than FNA for diagnosing MTC [6]. Several studies have demonstrated the usefulness of calcitonin in the diagnostic work-up of thyroid nodules [5,7,8]. In a series where > 10,000 patients with thyroid nodules underwent calcitonin testing, 0.4% had an elevated serum calcitonin level [8]. However, the direct screening measurement of serum calcitonin in all nodules with benign ultrasonic presentation is still a matter of debate due to the low prevalence of the disease [1]. Because of these limitations, many MTCs are still found incidentally after thyroidectomy, leading to the risk of incomplete treatment and thus of a poorer prognosis [9,10].

Ultrasound (US) is the preferred method for not only discriminating between benign and malignant thyroid lesions but also as guidance for FNA. In many established guidelines, nodules have been stratified on the basis of suspicious ultrasound characteristics [11-13]. However, many MTC nodules were not stratified by any system as having characteristics indicating a high risk of malignant tumours, and because of large size thresholds, fewer biopsies have been recommended [14,15]. Matrone et al. showed that FNA was only recommended for 49–64% of MTC nodules. Many MTCs without suspicious ultrasound features are not categorized as high risk of malignancy [15]. Vascularity, as an important characteristic of MTC, was not included in many societal US risk stratification systems [11]. Previous studies have also shown that hypervascularity is more frequent in MTC by comparing its US features with those of papillary thyroid cancer [16,17]. In this regard, our purpose with this study was to more fully investigate the vascularity characteristics of MTC on ultrasound and help screen nodules to identify MTC.

Adding Reference

[1] Kaliszewski K, Ludwig M, Ludwig B, Mikuła A, Greniuk M, Rudnicki J. Update on the Diagnosis and Management of Medullary Thyroid Cancer: What Has Changed in Recent Years? Cancers (Basel). 2022;14:3643.
[2] Matrone A, Gambale C, Prete A, Elisei R. Sporadic Medullary Thyroid Carcinoma: Towards a Precision Medicine. Front Endocrinol (Lausanne). 2022;29;13:864253.
[3] Elisei R. Routine serum calcitonin measurement in the evaluation of thyroid nodules. Best Pract Res Clin Endocrinol Metab. 2008;22:941-53.
[4] Elisei R, Bottici V, Luchetti F, Di Coscio G, Romei C, Grasso L, Miccoli P, Iacconi P, Basolo F, Pinchera A, Pacini F. Impact of routine measurement of serum calcitonin on the diagnosis and outcome of medullary thyroid cancer: experience in 10,864 patients with nodular thyroid disorders. J Clin Endocrinol Metab. 2004;89:163-8.

**Methods
- Did each of the 69 patients only have a single MTC nodule in this study? As we know, a patient may have multiple MTC nodules, while other types of nodules can also appear in the same patient.**

Response: Thank you for the comments and that is really a point. As your following suggestions, we expand the sample size. The present study consisted of 116 thyroid nodules in 102 patients with pathological proved MTC lesions, of which 102 patients had single MTC nodule and the other 14 patients presented with two MTC nodules, which has been added in methods and results section (Lines 77; Line 112-113). 6 patients also appear papillary thyroid cancer. The nodules detected by ultrasound and subjected to pathology were matched to the excised nodules according to size and location.

**- In addition, authors should explain under which conditions did two radiologists review the scans. Were they given only those 69 MTC images or did they get mixed dataset with other diagnoses (PTC, benign)? In other words, didn´t an a priori probability (an expectation of an expert about distribution of the data) play role in the classification process?**

Response: Thank you for the comments. Two radiologists reviewed the imaging in the mixed dataset with the other nodules in benign nodule Group, which has been added in methods section (Lines 102-104).

**- Interestingly, authors mentioned it in the limitation session, saying "The main limitations of this study were the relatively small number of patients in the non-blinded retrospective study". The authors only collected 18 l-MTC. Why not spend more time expanding the sample size? Otherwise, the statistical power is not enough.**

Response: Thank you for the comments. As your following suggestions, we expand the sample size. Between January 2017 and April 2023, a total of 8709 patients who underwent thyroidectomy at our center were retrospectively reviewed. 116 MTC nodules met the inclusion criteria. 85 lesions were classified as h-MTC and 31 lesions as l-MTC. Result sections and Tables are modified as follows (Lines 112-136; Table 1; Table 2).

Results

The clinical and biochemical characteristics of patients with MTC

The present study consisted of 116 thyroid nodules in 102 patients with pathologically proven MTC lesions, of which 88 patients had a single MTC nodule and the other 14 patients presented with 2 MTC nodules. The patients were predominantly female (60/102, 58.8%). The mean age was 47.6 ± 15.5 years (range, 18–84 years). Three cases (2.6%) were MEN 2. According to the ultrasonic criteria for risk classification, 85 nodules (73.3%) were classified as h-MTC (high suspicion by ATA classification) and 31 nodules (26.7%) as l-MTC [(intermediate suspicion by ATA classification (n = 22, 71.0%); low suspicion by ATA classification (n = 9, 29.0%)]. There was no statistically significant difference in age (p = 0.078), sex (p=0.38), preoperative Ct level (p=0.88), or preoperative CEA level (p=0.75) between the two groups. Moreover, patients in the l-MTC group had a larger tumour size than those in the h-MTC group (p<0.001) [Table 1]. All l-MTC nodules were initially found by US. Since they were classified as intermediate/low suspicion by the ultrasound risk evaluation, 22/31 (71.0%) of the lesions were followed up for a period before FNA or surgery. Of the 31 cases with l-MTC, the indication for FNA or surgery included suspicious lymph node metastasis on preoperative ultrasound (14, 45.2%); enlarged tumour size (8, 25.8%); and measurement of serum CEA/CT level due to suspicious MTC features (increased intranodular hypervascularity) on ultrasound (9, 29.0%). Of the 85 cases of h-MTC, all patients underwent FNA or surgery due to high suspicion of malignant thyroid nodules on preoperative ultrasound.

Blood supply characteristics on US and differences between the L-MTC group and the benign group

Regarding the echogenicity of the nodules, 11 (35.5%) were solid, 20 (64.5%) had a mixed structure, 26 (83.9%) were hypoechoic, and 5 (16.1%) were isoechoic/hyperechoic. In the present study, we compared the blood supply characteristics on US of l-MTC disease with 62 benign lesions from 62 patients. According to the blood flow pattern of CHAMMAS, 4 (12.9%) l-MTC disease had perinodular blood flow greater than central flow (pattern III), and 27 (87.1%) l-MTC disease had central blood flow greater than perinodular flow (pattern IV). Statistical analysis revealed a significant relationship between pattern IV and l-MTC (P<0.05). With respect to the CHEN pattern of vascularity distribution, 31 (100.0%) were type IV for the l-MTC, 14 (22.6%) were type I, 19 (30.6%) were type II, 13 (21.0%) were type III and 16 (25.8%) were type IV for the benign nodules. More penetrating branching vascularity was found in the l-MTC group than in the benign nodule group (23/31, 74.2% vs. 5/59, 4.8%, P<0.001). Thus, type IV blood flow patterns were more frequent in the L-MTC group (P<0.05) [Table 2, Figure 2].

Table 1. Comparison of Clinical characteristics

| Variable | l-MTC | h-MTC | P |
| --- | --- | --- | --- |
| No. | 31 | 85 |  |
| Age, y (median) | 48.00 | 47.45 | 0.078 |
| Sex (female/male) | 17/14 | 51/34 | 0.38 |
| Tumor size (cm) | 2.15 | 1.37 | <0.001 |
| Preoperative Ct level (pg/mL) | 416.80 | 443.58 | 0.88 |
| Preoperative CEA level (pg/mL) | 43.98 | 38.23 | 0.75 |

Table 2. Sonographic characteristics of L-MTC compared with benign group

| Variable | L-MTC | Benign Group | P |
| --- | --- | --- | --- |
| Age, y (median) | 48.00 | 54.61 | 0.08 |
| Sex (female/male) | 17/14 | 14/28 | 0.88 |
| Tumor size (cm) | 2.15 | 2.07 | 0.79 |
| Echogenicity |  |  | 0.30 |
| Hyperechogenicity/Isoechogenicity | 5 (16.1%) | 16 (25.8%) |  |
| Hypoechogenicity | 26 (83.9%) | 46 (74.2%) |  |
| Cystic change (%) | 20 (64.5%) | 29 (46.8%) | 0.10 |
| ATA classification |  |  | 0.06 |
| Intermediate Suspicion | 22 (71.0%) | 31 (50.0%) |  |
| Low Suspicion | 9 (29.0%) | 21 (33.9%) |  |
| Very low Suspicion | 0 | 10 (16.1%) |  |
| Vascularity |  |  |  |
| CHAMMAS pattern ^[15]^ |  |  | <0.001 |
| Pattern II | 0 | 14 (22.6%) |  |
| Pattern III | 4 (12.9%) | 28 (45.2%) |  |
| Pattern IV | 27 (87.1%) | 20 (32.3%) |  |
| CHEN pattern ^[16]^ |  |  | <0.001 |
| Pattern I | 0 | 14 (22.6%) |  |
| Pattern II b | 0 | 19 (30.6%) |  |
| Pattern III c | 0 | 13 (21.0%) |  |
| Pattern IV a | 31 (100%) | 16 (25.8%) |  |
| Penetrating branching vascularity |  |  | <0.001 |
| YES | 23(74.2%) | 5 (4.8%) |  |
| NO | 8 (25.8%) | 59 (95.2%) |  |

**- As this is a retrospective study, radiologists were not blinded to each other. This issue represent a bias that obviously affects the accuracy evaluation.**

Response: Thank you for the comments. Given the retrospective nature of the study, radiologists were not blinded and static image was utilized during the review process, bias may exist. The above has been added in limitation section (Lines 178-179). While, two radiologists reviewed the imaging respectively in the mixed dataset with other diagnoses (benign nodules group). Reviewers were blinded to the patients' clinical data, pathological results, and each other's evaluations.

**- Line 100, "an" should be "and".**
Response: Thank you for the comments. We followed the suggestion and correct the word (Lines 91). **Results
- Line 126, What does "MEN 2 in 2/69 (2.9%) cases" mean?**
Response: Thank you for the comments. We modified the sentences as follows. Three cases (2.6%) are MEN 2 (Lines 114).

**Discussion
- The discussion section is too short. Please enlarge discussion including literature revision.
- The manuscript could be improved by clearly stating the findings and could also be greatly improved by improved syntax and organization.**
Response: Thank you for the comments. As your following suggestions, we expand the discussion part and make it more clearly by improved organization as follows.

Discussion

The present results showed that approximately 1/3 of MTCs were not categorized as high risk of malignancy based on societal US guidelines, which led to most of these MTC lesions being followed up for a period before treatment in clinical practice. As vascularity is an important characteristic of l-MTC disease, we discerned a sonographic blood supply feature, penetrating branching vascularity, that identified more than 70% of l-MTC in our series. Only 5% of our cases with benign nodules displayed penetrating branching vascularity features, supporting that this feature is a valuable sign of l-MTC. We also showed that more CHAMMAS IV patterns (central blood flow greater than perinodular blood flow) (89% vs. 39%) and CHEN IV patterns (penetrating vascularity) (100% vs. 44%) were found in l-MTC disease than benign nodules.

Ultrasound is first-line imaging for patients with suspected MTC. Researchers have evaluated the sonographic features of MTCs in several studies, and US is associated with a sensitivity of 75% and a specificity of 93% [21]. In all these studies, it was reported that MTCs may not have classical ultrasonic malignant features of thyroid nodules, which include irregular shape, height/width ratio <1, and microcalcification. [22-24] In particular, patterns of benign features are often recorded in MTC, mainly including solid, oval to round shapes and smooth margins. Previous studies have shown that approximately one-third of MTC patients manifest as benign on ultrasound, which is very similar to the situation indicated by our data [6]. Although one study showed that the ATA guidelines performed well in the prediction of MTC [2], more studies and our data revealed that approximately 27%-47% of MTC cases were not classified by any of the systems as suggestive of a high risk of malignancy [15]. According to the ultrasonic criteria for risk classification, l-MTCs were misdiagnosed as such and were not treated early on. In our cases, most patients with those lesions (71%) were followed up for a period rather than given immediate treatment. Among our patients with l-MTC disease, indications for FNA or surgery included suspicion of lymph node metastasis on ultrasound, increased tumour volume, and serum CEA/CT levels. Calcitonin is more specific and sensitive than FNA for diagnosing MTC, while clinical screening of serum calcitonin is still not widely applied due to the low prevalence of the disease [1].

In addition to morphology, vascularity is also an important factor in the differential diagnosis of lesions. According to two previous studies, increased intranodular hypervascularity was more frequent in MTCs [16,17]. Our team’s previous study showed that hypervascularity is more frequent in medullary thyroid carcinoma by comparing it US features with those of papillary thyroid cancer [16]. However, Trimboli et al. [25] reported that 25% of MTCs exhibited an intranodular vascular signal. This discrepancy might be explained by the small sample size in the study and the inclusion of more h-MTCs. To the best of our knowledge, reports of the vascular pattern of l-MTCs in the literature are limited. In this study, we discovered the usefulness of penetrating branching vascularity in distinguishing l-MTCs. Penetrating branching vascularity, branching signals that extend from the flow of outside to the inside of the lesion, are different from intranodular vessels. The occurrence, development, and invasion of tumours highly depend on angiogenesis, which refers to the formation of new blood vessels through "endothelial cell germination" from the original microvascular network. Folkman et al. proved that proangiogenic factors generated from masses stimulated new blood vessels converging on lesions from some distance [26]. In our study, more than 70% of l-MTC cases manifested penetrating branching vascularity, demonstrating the value of penetrating branching vessels in the diagnosis of l-MTC disease. Moreover, the value of penetrating vessels in the diagnosis of malignant nodules was previously reported with the finding that thyroid cancer manifested penetrating vascularity [20,27]. Chen et al. showed that 40% (17/43) of malignant thyroid nodules manifested penetrating vascularity and that this feature could improve the diagnostic performance of TI-RADS for TR4 thyroid nodules [20]. Researchers at our centre also found previously that penetrating vessels around the lesion are useful for determining thyroid carcinoma [27].

The main limitation of this research was the relatively small number of patients in the retrospective study. Given the retrospective nature of the study, radiologists were not blinded, and static images were utilized during the review process. Bias may exist, and static images may not adequately reflect the status of the nodule. Prospective studies with larger sample sizes are necessary to validate our proposed vascularity feature. Second, this was a single-centre study. There might have been selection bias. Future research should expand on our observations to determine if the described pattern appears in other pathologies and its rate of occurrence.

In conclusion, based on societal US guidelines, some MTCs are mistakenly not categorized as high risk of malignancy, and vascularity can help diagnose l-MTC disease. We report a novel sonographic vascularity pattern of l-MTC, i.e., penetrating branching vascularity. The utilization of vascularity will help to identify MTC among nodules with low-intermediate suspicion by ultrasound risk classification to ensure appropriate clinical management.

**References
- Reference number 9 is repeated at point 19, and number 11 is repeated at point 24. Please thoroughly revise reference list following author's guidelines.**
Response: Thank you for the comments. We modified the reference and highlighted in red color (Lines 215-280). **Figures
- I have found that Figure 2D is not a color Doppler flow imaging, but a power Doppler imaging. Please replace it.**

Response: Thank you for the comments. We replaced the Figure 2D as follows.

We earnestly appreciate your critique and hope that the corrections will be met with approval. Thank you again for your careful reading and helpful comments.

Thank you very much

Yours, sincerely

Yu Xia
